# Pathogen-Specific Benefits of Probiotic and Synbiotic Use in Childhood Acute Gastroenteritis: An Updated Review of the Literature

**DOI:** 10.3390/nu15030643

**Published:** 2023-01-27

**Authors:** Maria Oana Săsăran, Cristina Oana Mărginean, Heidrun Adumitrăchioaiei, Lorena Elena Meliț

**Affiliations:** 1 Department of Pediatrics III Faculty of Medicine in English, George Emil Palade University of Medicine, Pharmacy, Science, and Technology of Târgu Mureș, Gheorghe Marinescu Street No 38, 540136 Târgu Mureș, Romania; 2 Department of Pediatrics I, George Emil Palade University of Medicine, Pharmacy, Science, and Technology of Târgu Mureș, Gheorghe Marinescu Street No 38, 540136 Târgu Mureș, Romania; 3 Doctoral School of Medicine, George Emil Palade University of Medicine, Pharmacy, Science, and Technology of Târgu Mureș, Gheorghe Marinescu Street No 38, 540136 Târgu Mureș, Romania

**Keywords:** probiotics, synbiotics, rotavirus infection, bacterial diarrhea, children

## Abstract

Probiotics represent viable microorganisms which are found within the normal gut microbiota, that exert strain-specific benefits in the management of several gastrointestinal disorders in children, including acute gastroenteritis. This review aims to evaluate the pathogen-specific role of probiotic supplementation in childhood diarrhea. A search of scientific databases was conducted to identify studies which investigated efficacy of probiotics and synbiotics in influencing outcome of acute gastroenteritis of known etiology. We identified 32 studies, most of which analyzed impact of probiotic supplementation in rotavirus gastroenteritis, while a very limited number of these conducted a separate analysis on bacterial diarrhea. *Lactobacillus rhamnosus* (*L. rhamnosus*), *L. reuteri* and *S. boulardii* still remain the most researched strains, with a proven role in decreasing diarrhea and hospitalization duration, especially in the setting of rotavirus infection. Combined products containing at least one of the aforementioned strains also performed similarly and might also influence rotavirus fecal shedding. Rotavirus immunization status has also been proposed as a significant influencing factor of probiotic use impact. The paucity of research focusing on bacterial etiologies, as well as of clinical trials conducted within ambulatory care units leaves room for further research on the matter, which needs to include larger cohort studies.

## 1. Introduction

Probiotics have been defined as oral supplements which contain viable microorganisms, in the form of bacteria and yeasts similar to those found within the microbiota of the normal, healthy gut [1,2]. The first evidence available in the literature regarding their existence comes from Henry Tissier, who was the first person to remark on a poorer stool bacterial culture in children infected with diarrhea, as well as in those fed with formula, as opposed to healthy and breastfed infants [3,4]. Since the publication of these first data in 1907, many studies have emerged, but their poor design and inadequate cultivation of bacteria in substrates other than human milk initially hindered the acquisition of reliable evidence. However, later research, and the ability to isolate and characterize specific bacterial cultures, showed the numerous health benefits of probiotics, which have been proven to improve intestinal health, alleviate symptoms related to lactose intolerance and decrease the risk of developing disorders such as inflammatory bowel disease, infectious diarrhea or allergies [3,5]. Thus, their administration in adequate amounts can be beneficial for the host and their lack of side effects has encouraged their widespread use [6].

Ensuring the equilibrium and integrity of intestinal microflora facilitates a functioning intestinal mucosal defense system, which in turn will trigger an appropriate immune response of the intestinal mucosa after exposure to non-self-antigens [7]. Probiotic products can interfere with the ability of invasive species to interact with endothelial receptors, can positively impact the integrity of enterocyte tight junctions, can stimulate local mucin production (Lactobacillus species) or the production of lactic acid and hydrogen peroxide which lowers intraluminal pH [8,9]. Thus, probiotic strains not only create a hostile environment for potential pathogens, but also enhance protection of the intestinal mucosa from external aggression. Probiotic effects vary from one strain to the other. For example, *Saccharomyces boulardii (S. boulardii)* is one of the protease-producing probiotics which promote neutralization of toxins such as those produced by *Escherichia coli* (*E. coli*), *Clostridium difficile* or *Vibrio cholerae* [10]. Lactobacilli species, on the other hand, produce β-galactosidase, an enzyme with a proven role in preventing diarrhea and facilitating lactose digestion [11].

As ongoing research provided insights into the role of probiotics in modulating mucosal immune response and combating antigen invasion, multiple randomized controlled trials assessed the role of probiotics in decreasing severity and duration of diarrhea-related symptoms and results obtained have constituted the basis of meta-analysis publications [12,13,14]. Most of these published trials have included pediatric populations, as diarrhea represents a major health burden at young ages, constituting one of the leading causes of morbidity and mortality under the age of 5 years [15]. The strains which were most frequently analyzed were *Lactobacillus rhamnosus GG* (*L. rhamnosus GG*) and *S. boulardii*, which also constitute the two major probiotics universally recommended through consensus statements, alongside with *L. reuteri*, in the management of acute gastroenteritis in children for decreasing symptom intensity and duration [16]. European guidelines sustain the use of *L. rhamnosus GG* and *S. boulardii* for 5–7 days, as adjuvant to oral rehydration solutions in childhood acute gastroenteritis, after consistent expert agreement, whereas American guidelines sustain the use of probiotic preparations in both infectious and antimicrobial-related diarrhea in children and adults, but only based on weak and moderate evidence [16,17]. Moreover, an update on the treatment of childhood acute gastroenteritis provided in 2020 by the Working Group on Probiotics and Prebiotics of the European Society for Paediatric Gastroenterology, Hepatology and Nutrition (ESPGHAN) also sustained the use of a combination between *L. rhamnosus* and *L. reuteri* and recommended against the simultaneous use of *L. helveticus* and *L. rhamnosus*, as well as against the use of *Bacillus clausii* (*B. clausii)* strains [18]. Thus, in spite of multiple probiotic strains available on the market, only a few have demonstrated their superiority in decreasing symptom intensity and duration of acute gastroenteritis compared to placebo and are as a result recommended as adjuvant therapies.

The beneficial probiotic effects seem to be strain specific, not only in the setting of acute gastroenteritis, but also in the prevention of antibiotic-associated diarrhea, the management of infantile colic or the treatment of other digestive tract conditions, such as *Helicobacter pylori* gastritis [19,20]. However, there are few reports available regarding the pathogen-specific benefits of probiotic strains, with a particular focus given in recent years to the outcome of probiotic supplementation upon the evolution and severity of diarrheic episodes with known etiology [21,22].

This review aims to investigate the pathogen-specific role of probiotic and synbiotic supplementation in pediatric acute gastroenteritis, in light of recent literature data.

## 2. Methods

We searched the Pubmed, Web of Science and Google Scholar databases for randomized controlled trials and pediatric population-based studies which have evaluated efficacy of probiotics and synbiotics in influencing outcome of acute gastroenteritis. We only focused on those studies which included patients with a known infectious etiology of the diarrheic disease or which had also performed a separate analysis on subgroups in which a certain viral or bacterial causative agent was identified or on those cohorts in which rotavirus vaccination status was known. We refrained from reporting data provided by publications limited to abstract formats or by in-extenso manuscripts written in a language different from English. Search terms used included “probiotic” AND “diarrhea” AND “child”, or “probiotic” AND “gastroenteritis” AND “child” or “probiotic” AND “rotavirus” or “prebiotic” AND “diarrhea” AND “child”, or “prebiotic” AND “gastroenteritis” AND “child” or “prebiotic” AND “rotavirus” or “synbiotic” AND “diarrhea” AND “child”, or “synbiotic” AND “gastroenteritis” AND “child” or “synbiotic” AND “rotavirus”.

## 3. Results

We identified 32 studies which complied with our inclusion and exclusion criteria, as summarized in Table 1, Table 2, Table 3 and Table 4. Most of these studies assessed the influence of probiotics on rotaviral diarrhea, whereas a very limited number of clinical trials evaluated the impact of probiotics upon bacterial diarrhea-related symptoms and aftermath. The most intensely researched probiotics on this subject still remain *L. rhamnosus*, *L. reuteri* and *S. boulardii*, but a few others have also showed promising potential in combating the burden of rotaviral or bacterial diarrhea-related hospitalizations and complications. In this paper, we will provide a background on probiotics and synbiotic products and detail how these impact the outcome of childhood acute gastroenteritis with an identified etiology.

### 3.1. Lactobacillus rhamnosus

*L. rhamnosus* has been proven to be one of the probiotics that can positively influence hospital stay length, as well as diarrhea duration (Table 1) [23]. A European multicenter trial, which randomized an impressive number of children aged 1 month to 3 years of age demonstrated that a combination of oral rehydration and *L. rhamnosus* is superior to oral rehydration alone in terms of reducing hospitalization duration and number of watery stools after 3 days of diarrhea evolution. Recovery from watery stools was significantly more rapid in those subjects whose stool samples were positive for rotavirus infection. Bacterial etiologies were scarcely detected, not allowing for a convenient statistical analysis, but no differences were reported in terms of diarrhea duration in these cases, independently of the study group allotment [24]. Likewise, a randomized controlled trial conducted in India highlighted the benefits of *L. rhamnosus* in decreasing diarrhea extent and timespan of parenteral rehydration, but only in those subjects confirmed with rotavirus infection, as opposed to other etiologies [25]. Similar results were reported by Guarino et al., with a slightly more consistent reduction of diarrhea duration in children diagnosed with rotavirus, as well as of fecal viral identification after 6 days of diarrhea onset [26]. Another study, conducted in Taiwan, suggested that *L. rhamnosus* also aids in decreasing the extent of fecal elimination of rotavirus, in a dose-dependent manner [27]. On the other hand, increase in stool consistency and recovery from diarrheic stools took place after a shorter amount of time with *L. rhamnosus* administration, but independently of the presence of rotavirus fecal shredding, according to the study of Aggarwal et al. [28]. Moreover, a meta-analysis reported a better performance of *L. rhamnosus* in children who had not received a vaccine against rotavirus and a lack of significant difference in gastroenteritis outcome in those who were immunized [29]. In terms of bacterial infections, data are scarce; one Indian study in which a little over 60% of the stool cultures were negative, but which individually assessed the role of *L. rhamnosus* in relation to each particular type of pathogen identified in the stool samples, concluded that its efficacy is limited to reducing diarrhea duration in *Clostridium difficile* positive patients. However, this conclusion is supported by a very limited number of cases [23].

Two randomized clinical trials which analyzed the efficacy of a combined probiotic strain containing *L. rhamnosus* and *L. helveticus* reported no virus-specific benefit over placebo in terms of reducing viral load at 28 days post-enrollment, nor in decreasing intensity of clinical symptoms. Both studies included children diagnosed with gastroenteritis of viral etiologies (norovirus, rotavirus, adenovirus), as well as of bacterial etiologies [21,22]. Still, a decrease in number of diarrhea episodes caused by adenovirus infection was noted (Table 1) [22].

There are limited reports regarding the efficacy of *L. rhamnosus* in the absence of hospitalization. An Indian study reported a significant reduction in diarrhea episodes during a follow-up period of 4 weeks in recipients of *L. rhamnosus* diagnosed with rotavirus gastroenteritis, as opposed to those with *Crytosporidial* diarrhea, in whom administration of the same probiotic showed no benefit compared to placebo. *L. rhamnosus* also seemed to augment specific IgG (and not IgA) production against rotavirus, but did not influence antibody levels for *Cryptosporidium* species (Table 1) [30]. Another randomized controlled trial, conducted in Uganda, highlighted the benefit of both *L. rhamnosus* and *S. boulardi* in reducing diarrhea episodes only in outpatient settings in children with severe acute malnutrition, and not in those who required hospitalizations. The study suggested that both strains do not influence the outcome and evolution of severe gastroenteritis, but provided no data regarding etiology of the diarrheic manifestations [31]. 

**Table 1:** Characteristics of clinical studies which assessed overall and pathogen specific impact of *L. rhamnosus* supplementation in childhood diarrhea.

Reference (Author, Year)	Type of Study	Population and Study Group Assignment	Type of Intervention			Main Outcome	Pathogen Specific Benefits
			Intervention/Study Group	Control Group	Duration of Treatment/Dosage		
Guandalini et al., 2000 [24]	Double-blind RCT	287 children, with ages: 1 month–3 years 147 in intervention group 140 in placebo group	*L. rhamnosus* + ORS	ORS	Dosage: 10^10^ CFU/250 mL 2×/days; up to 7 days	Reduction in duration of diarrhea and hospitalization period in the intervention group	Rotavirus positive patients: reduction in duration of diarrhea, in number of watery stools starting from the 3rd day of treatment and in hospitalization period in the intervention group Invasive pathogens—no significant differences between intervention and control group
Szymański et al., 2006 [25]	Double-blind RCT	87 children with ages 2 months–6 years 49 in intervention group 44 in control group	Three *L. rhamnosus* strains. (573L/1, 573L/2, 573L/3) + ORS/IV rehydration	ORS/IV rehydration	Dosage: 1.2 × 10^10^ CFU 2×/days, 5 days	No influence upon duration of diarrhea, of parenteral rehydration, nor of weight gain within the entire intervention group	Rotavirus positive patients: significant reduction of diarrhea duration, especially within the first 72 h of treatment; overall reduction in duration of parenteral rehydration
Guarino et al., 1997 [26]	Double-blind RCT	100 children with ages: 3–36 months 52 in intervention group 48 in control group	*L. rhamnosus* + oral rehydration therapy	Oral rehydration therapy	Dosage: 3 × 10^9^ CFU in 200 mL milk or formula, 2×/days, up to 5 days	Significant reduction in diarrhea duration in the intervention group versus control group	Rotavirus positive patients: slightly greater efficacy of *L. rhamnosus* in reducing diarrhea duration; significant reduction in viral excretion 6 days after the onset of diarrhea in the intervention group
Fang et al., 2009 [28]	RCT	23 patients with ages: 9–72 months 9 in the low-dose group, 8 in the high-dose group, 6 in control group	low-dose group and high-dose group—*L. rhamnosus*+ Simethicone	Simethicone 80 mg/day	Dosage: low-dose group—2 × 10^8^ *L. rhamnosus +* Simethicone 80 mg/day high-dose group—6 × 10^8^ *L. rhamnosus +* Simethicone 80 mg/day, 3 days	Significant reduction in fecal rotavirus concentration in the high-dose group after 3 days of treatment No significant difference in fecal rotavirus shedding in the low-dose and control group after 3 days	The study included only rotavirus positive patients
Aggarwal et al., 2014 [28]	Open Label RCT	200 children with ages: 6 months–5 years 100 in intervention group 100 control group	*L. rhamnosus* + Standard manage-ment (ORS/IV rehydration + zinc 20 mg/day for 14 days)	Standard management	Dosage: 10^10^ CFU once daily, 5 days	Significant reduction in duration of diarrhea and in time to improvement in stool consistency	Significant reduction in duration of diarrhea and in time to improvement in stool consistency independently of the presence/absence of Rotavirus fecal antigen
Basu et al., 2007 [23]	Double-blind RCT	235 children with ages: 2–6 years 117 in intervention group 118 control group	*L. rhamnosus* + ORS	ORS	Dosage: 60 million cells dissolved in ORS twice daily, at least 7 days/until diarrhea stopped	Significant reduction in duration of diarrhea and hospital stay	*C. difficile* positive patients: Significant reduction in duration of diarrhea
Freedman et al., 2020 [21,22]	Double-blind RCT	816 patients with ages: 3–48 months 408 in intervention group 408 control group	*L. rhamnosus RO011 + L. helveticus RO052* (95:5 ratio) + standard therapy	Standard therapy	Dosage: 4 × 10^9^ CFU *L. rhamnosus RO011 + L. helveticus RO052*(95:5 ratio) + standard therapy twice daily, 5 days	No significant changes in disease severity, assessed using the modified Vesicari score [32]	No significant reduction in stool viral and bacterial load
Freedman et al., 2022 [22]	Two Double-blind RCT	1565 patients with ages: 3–48 months 778 in intervention group 787 control group	US Group—370 with *L. rhamnosus GG* Canadian Group—408 *L. rhamnosus* RO011 + *L. helveticus* RO052 (95:5 ratio) + standard therapy	Standard therapy	Dosage: US Group—received only *L. rhamnosus*—10^10^ CFU, twice daily, 5 days Canadian Group—4 × 10^9^ CFU *L. rhamnosus RO011 + L. helveticus RO052* (95:5 ratio) + standard therapy twice daily, 5 days	No significant changes in disease severity, assessed using the modified Vesicari score [32]	No significant reduction in stool viral and bacterial load Adenovirus positive patients: significant decrease in number of diarrhea episodes in those supplemented with *L. rhamnosus* RO011 + *L. helveticus* RO052
Sindhu et al., 2014 [30]	Double-blind RCT	124 children with ages: 6 months–5 years (82 with *Rotavirus* and 42 with *Cryptosporidium* Species) 65 in intervention group 59 in control group	*L. rhamnosus GG* + antibiotic treatment (in case of positive stool culture)	Placebo + antibiotic treatment (in case of positive stool culture)	Dosage: 10^10^ CFU once per daily for 4 weeks	No influence of the probiotic product upon diarrhea duration, severity, fever, frequency of vomiting episodes, dehydration status or hospital stay	Rotavirus positive patients: significant reduction in diarrhea episodes, augmentation of specific IgG antibody production against rotavirus *Cryptosporidium* positive patients: no influence upon diarrhea duration compared to placebo; no impact on specific antibody levels

Legend: CFU—colony forming units; *C. difficile*—*Clostridium difficile*; IV—intravenous, *L. helveticus—Lactobacillus helveticus*; *L. rhamnosus—Lactobacillus rhamnosus*; ORS—oral rehydration solution; RCT—randomized controlled trial.

### 3.2. Saccharomyces boulardii

*S. boulardii* has been regarded as the most effective agent in reducing duration of diarrhea (including the risk of an evolution of loose stools over 2 days) and is apparently superior to other strains such as *B. lactis*, *L. rhamnosus*, *L. reuteri* and *L. paracasei*, according to a meta-analysis conducted on 84 studies [33]. Moreover, its advantages over *B. clausii* in shortening diarrhea extent have been highlighted within a randomized trial, which also showed comparable performance of the two strains in terms of hospitalization, fever duration and number of vomiting episodes [34]. These results contradict former scientific reports such as that of Gaon et al., which claimed that *S. boulardii*’s efficacy is similar to the efficacy of a combination between *L. casei* and *L. acidophilus* in diminishing diarrhea and vomiting, when compared to placebo and is independent of Rotaviral etiology (Table 2) [35].

Table 2 details characteristics and results of studies which analyzed overall and pathogen-specific impact of *S. boulardii* in pediatric gastroenteritis. One randomized controlled trial conducted in India certified the advantages of *S. boulardii* over a similar product in rotavirus-associated diarrhea, without any adverse events, but only in terms of hospitalization duration. No difference from controls was seen in terms of fever duration, vomiting episodes, proportion of children requiring parenteral rehydration or with diarrhea lasting for more than one week [36]. Similarly, another study conducted by Dalgic et al. established a significant decrease in duration of hospitalization and diarrhea in children diagnosed with rotavirus infection, supplemented with both *S. boulardii* and zinc, as opposed to controls. Moreover, the authors also demonstrated that this adjuvant therapeutic combination can also accentuate resolution of vomiting [37]. On the other hand, a Bolivian randomized controlled trial reported that number of recurrent vomiting episodes is decreased in patients receiving an oral rehydration therapy in combination with *S. boulardii*, *B. longum*, *L. rhamnosus* and *L. acidophilus* as opposed to those which only benefited from the addition of *S. boulardii* in their treatment or who received placebo together with oral rehydration solutions (ORS). Still, *S. boulardii* alone showed a better outcome than the four-strain product and placebo in shortening the number of days with fever and diarrhea in children infected with rotavirus [38]. Apparently, a three-day treatment with *S. boulardii* performed even better in inhibiting release of watery stools in pre-school children who tested positive for rotavirus, unlike in those who were negative for the virus [39].

Persistent, prolonged elimination of rotavirus in feces has been reported even in vaccinated children and represents a source of community-acquired infection [40]. *S. boulardi* might be a potential inhibitor of rotavirus fecal shedding, with absence of fecal virus detection after 5 days of administration, as suggested by a multicenter, case-control clinical trial. This conclusion was, however, supported by a very limited number of cases [41].

**Table 2:** Characteristics of clinical studies which assessed overall and pathogen specific impact of *S. boulardii* supplementation in childhood diarrhea.

Reference (Author, Year)	Type of Study	Population and Study Group Assignment	Type of Intervention			Main Outcome	Pathogen Specific Benefits
			Intervention/Study Group	Control Group	Duration of Treatment/Dosage		
Gaón et al., 2003 [35]	Double-blind RCT	89 children with ages: 6–24 months 29 in control group (group 1). 30 in *S. boulardii* (group 2) 30 in *Lactobacilli* group (group 3)	Group 2: *S. boulardii* Group 3: Pasteurized cow milk with *L. casei* and *L. acidophilus*	Group 1: Pasteurized cow milk + ORS	Dosage: *S. boulardii* group 10^10^ CFU/g twice daily for 5 days Group with: Pasteurized cow milk with *L. casei* and *L. acidophilus*: 10^10^–10^12^ CFU/g twice daily for 5 days	Similar significant reduction in vomiting, diarrhea duration and number of stools in both study groups	No specific benefits in relation to the presence/absence of rotavirus fecal antigen
Das et al., 2016 [36]	Double-blind RCT	60 children with ages: 3 months–5 years 30 in intervention group 30 in control group	*S. boulardii*	Placebo	Dosage: 250 mg 2×/day for 5 days	Significant reduction in diarrhea duration and hospitalization period No influence on fever and vomiting duration nor on proportion of children who required parenteral rehydration or presented diarrhea lasting for >7 days	The study included only rotavirus positive patients
Dalgic et al., 2011 [37]	Single Blind RCT	480 children with ages: 1–28 months, divided into 7 intervention groups and 1 control group (60 patients included in each group)	Group 1: *S. boulardii* Group 2: zinc (Zn) Group 3: lactose-free formula (LF) Group 4: *S. boulardii* + Zn Group 5: *S. boulardii* + LF Group 6: Zn + LF, Group 7: Zn + LF + *S. boulardii* + ORS and/or parenteral rehydration	Group 8 (control group): −ORS and/or parenteral rehydration	Dosage: *S. boulardii*—250 mg once daily 5 days Zn—10 mg 2×/day in infants < 6 months; 20 mg 2×/day in infants/children > 6 months	Statistically significant decrease in diarrhea duration and hospitalization time in groups 2 and 4 versus controls	The study included only rotavirus positive patients
Grandy et al., 2010 [39]	Double-blind RCT	64 hospitalized children with ages: 1–23 months divided in 3 groups Group GC (control): 20 children Group GB: 21 children Group GARLB: 23 children	Group GB: *S. boulardii* + ORS Group GARLB: combined probiotic product (*L. acidophilus*, *L. rhamnosus*, *B. longum* and *S. boulardii)*	Placebo	Dosage: For *S. boulardii*—4 × 10^10^ lyophilized cells/dose 2×/day for 5 days For combined probiotic product—(*L. acidophilus* 6.625 × 10^7^ lyophilized cells/dose, *L. rhamnosus* 3.625 × 10^7^ lyophilized cells/dose, *B. longum* 8.75 × 10^6^ lyophilized cells/dose and *S. boulardii)* 1.375 × 10^7^ lyophilized cells/dose) 2×/day for 5 days	Statistically significant reduction in diarrhea duration in both study groups versus control; *S. boulardii* alone performed better than the combined product in this regard Significant reduction in diarrhea and vomiting duration when comparing the merged intervention groups versus placebo	The study included only rotavirus positive patients
Corrêa et al., 2011 [39]	Double-blind RCT	176 patients with ages: 6–48 months 90 in intervention group 86 in control group	*S. boulardii*	Placebo—excipients	Dosage: 4 × 10^9^ viable cells 2×/day for 5 days	Statistically significant decrease in frequency and duration of diarrhea if *S. boulardii* was administered during the first three days of gastroenteritis evolution	Rotavirus positive patients—statistically significant decrease in frequency of diarrhea after 3 days of intervention
Mourey et al., 2020 [41]	Double-blind RCT	100 children with ages: 3–36 months 49 in intervention group 51 in control group	*S. boulardii* + ORS + Zn	Placebo + ORS + Zn	Dosage: *S. boulardii* 5 × 10^9^ living cells 2×/day for 5 days Zn—10 mg 1×/day in infants < 1 year; 20 mg 1×/day in children > 1 year	Significant increase in stool consistency starting from day three of intervention Significant decrease in time of recovery from diarrhea in the intervention group	Rotavirus positive patients: absence of fecal virus detection after 5 days of treatment in old patients belonging to the intervention group

**Legend**: CFU—colony forming units; *L. acidophilus*—*Lactobacillus acidophilus*; *L. casei*—*Lactobacillus casei*; LF—lactose-free formula; ORS—oral rehydration solution; RCT—randomized controlled trial; *S. boulardii*—*Saccharomyces boulardii*; Zn—zinc.

### 3.3. Lactobacillus acidophilus

*L. acidophilus* has been one of the bacteria widely used alone or as part of combined products for the treatment of acute diarrhea in children, in spite of insufficient evidence available [2]. The suppositions surrounding the potential beneficial effects of this strain emerged from evidence supporting its involvement in inhibition of bacterial invasion of epithelial intestinal cells, a consistent characteristic of other Lactobacilli as well [42]. *L. acidophilus* can also augment specific immune reactions and is stable at high temperatures, so it is unsurprising that several compounds appeared on international markets, which claimed to deliver relief of gastroenteritis-associated symptoms [43]. However, one study and a meta-analysis reported no benefit regarding its complementary use in those pediatric gastroenteritis cases in which an etiological factor was identified from the analyzed stool specimens [44,45]. In similar fashion, one randomized controlled trial reported no clear advantage of *L. acidophilus* over placebo in cases where rotavirus or norovirus was identified, neither in terms of reducing symptom duration, nor in terms of diminishing stool viral load [46]. Some evidence emerged regarding the advantages of *L. acidophilus* in shortening hospitalization duration or number of days with fever, but only in patients with unknown etiology of the diarrhea or in whom a rotavirus infection was excluded, as detailed in Table 3 [44,47,48]. Salazar-Lindo et al. also reported a lower number of stools in those children supplemented with *L. acidophilus* suffering from mild diarrhea, but in their study cohort an infectious agent (namely rotavirus) was identified in only a very limited number of patients [49]. Given the inconsistent results reported by various studies, a meta-analysis conducted by authors belonging to the Working Group on Probiotics and Prebiotics of the ESPGHAN concluded that there is insufficient evidence available to support the administration of *L. acidophilus* in childhood acute gastroenteritis [50].

### 3.4. Lactobacillus reuteri

*L. reuteri* strains are able to modulate lipopolysaccharide-induced intestinal inflammation and reduce mucosal damage caused by enteric infections in rats [51]. Both anti-bacterial and anti-inflammatory effects have been associated with their use, as demonstrated in a murine model in which the administration of *L. reuteri* antagonized the extension of enteral mucosa inflammation and *C. difficile* infection [52]. Moreover, two in vitro studies also confirmed *L. reuteri*’s potential in combating *E. coli* growth [53], modulating immune response against *Salmonella typhimurium* [54] and another study conducted on rats related its use with the development of mucosal rotavirus-specific IgA production [55]. A randomized controlled trial conducted in pre-school children asserted the protective effect of *L. reuteri* against infectious diarrhea, which lasted through a follow-up period of 3 months after previous administration of the product for the same amount of time (Table 3) [56].

Shornikova et al. reported amelioration of diarrhea in a small pediatric cohort which received *L. reuteri*, as opposed to placebo, with most of the patients belonging to the intention-to-treat group not experiencing any watery stools after two days of probiotic supplementation. Given that rotavirus infection was identified in 75% of the study population, the authors proposed *L. reuteri* as a potential therapeutic adjuvant in rotavirus enteritis. However, the study failed to identify any significant increase in specific rotavirus IgA antibody titer in the *L. reuteri* recipient group [57]. Another research study by these authors, performed on a rotavirus cohort in children aged between 6 and 36 months, confirmed the significant reduction in the duration of watery diarrhea associated with *L. reuteri* administration, as well as a good restoration of the enteral microbiota in this setting (Table 3) [58].

A Botswanian pediatric randomized controlled trial which included subjects vaccinated against rotavirus found no significant association between the administration of *L. reuteri* and acute gastroenteritis outcome, whether viral or bacterial. No notable replication inhibition of bacteria such as *Shigella*, *Campylobacter*, Enteropathogenic *E. coli* or *Cryptosporidium* was reported, but the study did not separately evaluate the subpopulation in which enteric viral infections were found in feces samples, as most of those children also presented another viral/bacterial co-infection [59]. Moreover, a Polish study found no benefit of *L. reuteri* on diarrhea-related outcomes, regardless of rotavirus vaccination status, but the infectious etiology was unknown in most of the patients enrolled [60].

### 3.5. Lactobacillus plantarum

Lactic acid bacteria have the ability to attach to cellular monolayers, exerting protection against rotavirus, as well as against the transmissible gastroenteritis virus (TGEV), according to Maragkoudakis et al. [61]. Out of all the Lactobacillus species studied, *L. plantarum* had the highest attachment ability and was found to have an antiviral effect against both viruses, as proven by the same experimental study [61]. Another study performed on mice proved that exopolysaccharides released by *L. plantarum* effectively reduce the number of diarrheic episodes and rotaviral shedding [62]. The decrease in rotavirus load in subjects supplemented with a *L. plantarum* product was later confirmed by research conducted in children, which also showed a significant decrease in diarrhea duration and in its severity (assessed through daily changes in Vesikari score—Table 3 [32]) [63].

**Table 3:** Characteristics of clinical studies which assessed overall and pathogen specific impact of probiotic *L. acidophilus*, *L. reuterii* and *L. plantarum* in childhood diarrhea.

Reference (Author, Year)	Type of Study	Population and Study Group Assignment	Type of Intervention			Main Outcome	Pathogen Specific Benefits
			Intervention/Study Group	Control Group	Duration of Treatment/Dosage		
Pinto et al., 2016, 2016 [44,47]	Retrospective cohort study	290 children with ages: 6–60 months 65 in study group 225 in control group	*L. acidophilus* mixture (80% *L. acidophilus*, 10% *L. bulgaricus*, 5% *B. bifidum*, 5% *S. thermophilus*	Standard treatment	Dosage: 1 × 10^9^ CFU/g 2×/day (no information regarding duration of treatment)	Significant decrease in diarrhea duration of IV rehydration among the study group No influence of the probiotic supplementation upon length of hospital stay	Patients with positive stool studies (positive stool culture, positive stool for *C. difficile* toxin, positive rotavirus antigen)—no benefits from probiotic use as opposed to those with negative stool studies
Khanna et al., 2005 [46]	Double-blind RCT	98 children with ages: 6 months–12 years 48 in intervention group 50 in control group	*L. acidophilus* + ORS	ORS	Dosage: 1.5 × 10^10^ tyndalized cells 1×/day for 3 days	No significant benefit of probiotic products in reducing mean diarrhea duration, mean ORS requirement, mean rehydration period and duration of hospital stay	Rotavirus positive patients—no beneficial effect in the intervention group
Hong Chau et al., 2018 [46]	Double-blind RCT	290 children with ages: 9–60 months 143 in intervention group 147 in control group	*L. acidophilus* + ORS + Zn + antimicrobials were needed	Placebo + ORS + Zn + antimicrobials were needed	Dosage: 1 × 10^8^ CFU cells 2×/day for 5 days	No significant reduction in diarrhea and hospitalization duration, nor in stool frequency	Rotavirus/Norovirus positive patients—no significant decrease in stool load and no influence of intervention upon symptom duration
Shornikova et al., 1997 [57]	Double-blind RCT	40 children with ages: 6–36 months 19 in intervention group 21 in control group	*L. reuteri* + ORS	Placebo + ORS	Dosage: 10^10^–10^11^ CFU/g 1×/day for 5 days/hospitalization period (if shorter than 5 days)	Shorter mean duration of diarrhea during hospital stay in the intervention group No influence on dehydration correction and electrolyte levels of the probiotic supplementation Significant reduction in vomiting after the second day of treatment in the intervention group	No influence of the intervention treatment upon rotavirus IgA antibody production
Shornikova et al., 1997 [58]	Double-blind RCT	66 children with ages: 6–36 months 20 in group 1 21 in group 2 25 in control group	Group 1: L. reuteri—small dose + ORS Group 2: *L. reuteri*—large dose + ORS	Placebo + ORS	Dosage: Small dose: 2.5 × 10^6^–5 × 10^6^ CFU/mL 1×/day for a maximum of 5 days Large dose: 5 × 10^8^–2.5 × 10^9^ CFU/mL 1×/day for 5 days	Large dose of *L. reuteri* yielded a significant reduction in duration and frequency of diarrhea after 2 days of treatment Good restoration of enteral microbiota in both intervention groups	The study included only rotavirus positive patients No influence of *L. reuteri* upon rotavirus IgA and IgG antibody titers
Pernica et al., 2022 [59]	Multicentric double-blind RCT	272 children with ages: 2–60 months 135 in intervention group 137 in control group	*L. reuteri* + ORS/parenteral IV rehydration + antibiotic treatment (were needed)	Placebo + ORS/parenteral IV rehydration + antibiotic treatment (were needed)	Dosage: 1 × 10^8^ CFU/g 1×/day for 60 days	No significant impact of the probiotic strain upon recurrence of diarrhea, length of hospital stay, developmental of sepsis, 7-day and 60-day mortality rate during the follow-up period of 60 days	Patients with positive stool culture for *Shigella*, *Campylobacter*, enteropathogenic *E. Coli* or *Cryptosporidium*: no notable replication inhibition of the identified bacteria
Szymański et al., 2019 [60]	Double-blind RCT	91 children with ages: <5 years 44 in intervention group 47 in control group	*L. reuteri* + standard rehydration therapy	Placebo + standard rehydration therapy	Dosage: 2 × 10^8^ CFU 1×/day for 5 days	Significant decrease in duration of hospitalization in the intervention group No influence of intervention therapy upon diarrhea duration, frequency and recurrence, duration of IV rehydration, nor upon improvement of Vesikari scale	Children not vaccinated against rotavirus—significant reduction in hospital stay in the intervention group; no influence of intervention therapy upon diarrhea duration Children vaccinated against rotavirus—similar primary and secondary outcome in both groups
Shin et al., 2020 [63]	RCT	50 children with ages: 1–69 months 15 in group 1 8 in group 2 27 in group 3	Group I: *L. plantarum* LRCC5310 Group 3: *Saccharomyces* species	Group 2: Placebo + standard treatment	Dosage: not specified	Significant decrease in stool frequency and diarrhea duration between group I and placebo in day 3 of hospitalization Significant decrease in diarrhea duration and in changes of Vesikari scale when comparing merged intervention groups with placebo	The study included only rotavirus positive patients Significant reduction in rotavirus fecal titer in group 1 versus placebo

**Legend**: CFU—colony forming units; IV—intravenous, *L. acidophilus*—*Lactobacillus acidophilus*; *L. plantarum*—*Lactobacillus plantarum*; *L. reuteri*—*Lactobacillus reuteri*; ORS—oral rehydration solution; RCT—randomized controlled trial; *S. thermophilus*—*Streptococus thermophilus*; Zn—zinc.

### 3.6. Combinations of Multiple Probiotic Strains

Table 4 provides insights regarding the specific beneficial use of combined probiotic products in childhood diarrhea. A double-blind randomized controlled trial reported effective reduction of diarrhea duration in children who were administered twice daily a combined suspension, consisting of 6 different strains: *B. longum*, *B. lactis*, *L. acidophilus*, *L. rhamnosus*, *L. plantarum* and *Pediococcus pentosaceus*. The study also established that each individual component of the combined aforementioned suspension can inhibit in vitro rotavirus infection, without cytotoxic effects on the population of cells analyzed [64]. A *B. longum* and *L. acidophilus* mixture can also significantly shorten diarrhea duration, according to a Korean study [65]. Another similar study demonstrated significant reduction in hospitalization duration in children infected with rotavirus who received a mixed probiotic preparation consisting of *L. acidophilus*, *L. rhamnosus*, *B. longum* and *S. boulardii* for at least 5 days as opposed to oral/parenteral rehydration alone [66]. A preparation containing *Bacillus mesentericus*, *Clostridium butyricum* and *Enterococcus faecalis* also seems to effectively decrease diarrhea severity in combination with symptomatic and rehydration therapy after 3 days of daily two-dose administration in patients infected with rotavirus, but not in those infected with *Salmonella* [67]. Similarly, the administration of a suspension containing *L. casei*, *L. rhamnosus*, *Streptococcus thermophilus* (*S. thermophilus*), *B. breve*, *L. acidophilus*, *L. bulgaricus* and *B. infantis* twice daily for a minimum of 5 days reduced the number of hospitalization days related to rotaviral diarrhea [68]. A combination of 8 probiotic strains (*L. acidophilus*, *L. paracasei*, *L. bulgaricus*, *L. plantarum*, *Bifidobacterium breve*, *Bifidobacterium infantis*, *Bifidobacterium longum* and *S. thermophilus*) has also been proposed as an adjuvant therapeutic agent in rotavirus diarrhea, as it promoted a shorter recovery time and significantly decreased the requirement of oral rehydration therapy and stool volume loss after just two days of administration [69]. Furthermore, the simultaneous administration of *L. rhamnosus* and *L. reuteri* supposedly reduces the detection rate of rotavirus fecal antigen after 5 days of treatment, which suggests that these two probiotics positively interfere with viral elimination time. Still, this evidence is supported by a study with very limited follow-up time [70].

### 3.7. Synbiotic Products

Prebiotic supplements in the form of pectin-derived acidic oligosaccharides, short-chain galacto-oligosaccharides, long-chain oligosaccharides added to a fermented milk concentrate have been proven to enhance protection against rotavirus infection in suckling rats, through a presumed interaction with viral particle attachment to the enterocyte, which consequently inhibits viral replication [71,72]. Synbiotic products, containing both prebiotics and probiotics seem to also stimulate immune response against enteric viruses through modulation of the interferon signaling pathway [73]. Hence, synbiotics have been proposed as additional products to classical therapy in severe rotavirus enteritis by a review mainly based on in vitro studies [74]. The authors acknowledged, however, that the paucity of randomized controlled trials mandated future research investigating the preventive and therapeutic benefits of synbiotics in rotavirus diarrhea [74].

Evaluation of etiology-based performance of synbiotics in the management of diarrhea in children has been so far based on two randomized-controlled trials, one of them including only patients in whom stool samples were positive for rotavirus and the other one conducting a separate analysis on children with diarrhea triggered by the same etiological agent [75,76]. As detailed in Table 4, Dewi et al. proved that a combination of *L. casei*, *L. rhamnosus*, *S. thermophilus*, *B. breve*, *L. acidophilus*, *B. infantis* and *L. bulgaricus* and fructo-oligosaccharides (FOS) can aid in decreasing diarrhea duration in children with rotaviral gastroenteritis, whereas Islek et al. reported the augmentation of the same shortening in diarrhea timespan in the study subgroup with rotavirus infection in which *B. lactis* together with inulin were administered, as opposed to children infected with adenovirus or *Entamoeba histolytica*, in whom the synbiotic produced no significant difference [75,76]. Another three studies have also supported products consisting of multiple probiotic strains and FOS as complementary agents to standard treatment that can yield a shorter recovery of stool consistency and overall decrease in duration of diarrhea [77,78,79]. Moreover, *L. paracasei* combined with arabinogalactan and xylo-oligosaccharides can improve stool output frequency, positively impact hospital stay and decrease parental working days absences, according to an Australian study [80]. However, all these four studies have provided no information regarding diarrhea etiology for any of the pediatric patients enrolled [77,78,79,80].

**Table 4:** Characteristics of clinical studies which assessed overall and pathogen specific impact of combined probiotic strains and synbiotic supplementation in childhood diarrhea.

Reference (Author, Year)	Type of Study	Population and Study Group Assignment	Type of Intervention			Main Outcome	Pathogen Specific Benefits
			Intervention/Study Group	Control Group	Duration of Treatment/Dosage		
Lee et al., 2015 [64]	Double-blind RCT	29 children with ages: 3 months–7 years 13 in intervention group 16 in control group	Combined product: *B. longum*, *B. lactis*, *L. acidophilus*, *L. rhamnosus*, *L. plantarum* and *Pediococcus pentosaceus*	Placebo	Dosage: 10^9^ CFU/g (10^8^ CFU/each strain) 1×/day for 1 week	Significant decrease in diarrhea duration after 7 days of treatment No significant difference in abdominal pain, vomiting, fever duration, nor in paraclinical data between the two groups at the end of treatment	Rotavirus positive patients—significant decrease in diarrhea and vomiting duration after 7 days of treatment; no significant difference in abdominal pain, fever duration, nor in paraclinical data between the two groups at the end of treatment
Park et al., 2017 [65]	Double-blind RCT	57 children with ages: 9–16 months 28 in intervention group 29 in control group	Combined product: *B. longum*, *L. acidophilus*	Placebo	Dosage: 2.2 × 10^9^ CFU/g of probiotic mixture of *B. longum* 2 × 10^10^ CFU/g and *L. acidophilus* 2 × 10^9^ CFU/g 2×/day for 3 days	Significant reduction in diarrhea duration in the intervention group No impact of the combined product upon fever duration, diarrhea and vomiting frequency	The study included only rotavirus positive patients
Teran et al., 2009 [66]	Single-blind RCT	75 children with ages: 28 days–24 months 25 in group 1 (nitazoxanide group) 25 in group 2 (probiotic group) 25 in control group	Group 1: Nitazoxanide Group 2: combined product—*L. acidophilus*, *L. rhamnosus*, *B. longum* and *S. boulardii*	Standard treatment	Dosage: Nitazoxanide—15 mg/kg/day divided into 2 doses for 3 days Combined product—1.25 × 10^8^ CFU/g 2×/day for 5 days	Significant reduction in duration of diarrhea and hospitalization period in both intervention groups versus placebo	The study included only rotavirus positive patients
Huang et al., 2014 [67]	Open Label RCT	159 children with ages: 3 months–14 years 82 in intervention group 77 in control group	Combined product: *E. faecalis*, *C. butyricum*, *B. mesentericus*	Suportive treatment	Dosage: a tablet of the combined probiotic product contains: 3.48 × 10^8^ CFU of a mixture of *E. faecalis* (3.17 × 10^8^ CFU), *C. butyricum* (2 × 10^7^ CFU) and *B.mesentericus* (1.1 × 10^7^ CFU) children < 6 years—2 × 1 tabl/day children 6–12 years—2 × 2 tabl/day children > 12 years—2 × 3 tabl/day for 7 days	Significant reduction in diarrhea duration and Vesikari score in the intervention group No influence of intervention therapy upon mild stool frequency and hospitalization duration	Rotavirus positive patients: significant reduction in diarrhea severity after 3 days of probiotic treatment Salmonella positive patients: no significant reduction in diarrhea severity in patients belonging to intervention group
Sobouti et al., 2016 [68]	Single-blind RCT	60 patients with ages: 3 months–7 years 32 in intervention group 28 in control group	Combined product: *L. casei*, *L. rhamnosus*, *S. termophilus*, *B. breve*, *L. acidophilus*, *L. bulgaricus*, *B. infantis* + ORS/IV rehydration therapy	ORS/IV rehydration therapy	Dosage: 1 × 10^9^ CFU/g 2×/day for 5 days	Significant decrease in hospitalization duration in the intervention group No association was found between probiotic treatment and degree of dehydration	The study included only rotavirus positive patients
Dubey et al., 2008 [69]	Double-blind RCT	224 children with ages: 6 months–2 years 113 in intervention group 111 in control group	Combined product: *L. acidophilus*, *L. paracasei*, *L. bulgaricus*, *L. plantarum*, *B. breve*, *B. infantis*, *B. longum*, *S. thermophilus* + ORS/IV rehydration therapy	Placebo + ORS/IV rehydration therapy	Dosage: each sachet of the combined probiotic product contains 9 × 10^10^ CFU/g children < 5 kg—2 sachets/day for 4 days children—5–10 kg—4 sachets/day for 4 days	Significant reduction in recovery time in the intervention group Significant decrease in stool frequency starting from the second day of treatment No significant difference between the two groups in term of volume of ORS/parenteral solutions administered	The study included only rotavirus positive patients
Rosenfeldt et al., 2002 [70]	Double-blind RCT	69 children with ages: 6–36 months 30 in intervention group 39 in control group	Combined product: *L. rhamnosus*, *L. reuteri* + ORS/IV rehydration therapy	Placebo + ORS/IV rehydration therapy	Dosage: 2.2 × 10^10^ CFU of a mixture of *L. rhamnosus* (1.7 × 10^10^ CFU), *L. reuteri* (0.5 × 10^10^ CFU) 2×/day for 5 days	Significant decrease in hospital stay duration, duration of diarrhea after start of treatment and in reduction of loose stool frequency on the 5^th^ day of follow-up in intervention versus control group No significant difference between the two groups in term of fever duration	Significant reduction in fecal rotavirus antigen positivity rate on day 5 among the intervention group
Dewi et al., 2015 [75]	Double-blind RCT	69 children with ages: 6–59 months 34 in intervention group 35 in control group	Synbiotic product: *L. casei*, *L. rhamnosus*, *S. thermophilus*, *B. breve*, *L. acidophilus*, *B. infantis*, *L. bulgaricus*, FOS + ORS/IV rehydration therapy + Zn	Placebo + ORS/IV rehydration therapy + Zn	Dosage: a sachet of the synbiotic product contains 1 × 10^9^ CFU/dose of a mixture of *L. casei* (4 × 10^8^ CFU), *L. rhamnosus* (3.5 × 10^8^ CFU), *S. thermophilus* (1 × 10^8^ CFU), *B. breve* (5 × 10^7^ CFU), *L. acidophilus* (5 × 10^7^ CFU), *B. infantis* (4 × 10^7^ CFU), *L. bulgaricus* (1 × 10^7^ CFU) and FOS (990 mg) 1×/day for 5 days	Significant decrease in diarrhea recovery time No impact upon hospital stay	The study included only rotavirus positive patients
Islek et al., 2014 [76]	Double-blind RCT	156 children with ages: 2–60 months 79 in intervention group 77 in control group	Synbiotic product: *B. lactis*, inulin + standard treatments	Placebo + standard treatment	Dosage: a sachet of the synbiotic product contains a mixture of *B. lactis* (5 × 10^10^ CFU) and inulin (900 mg) 1×/day for 5 days	Significant decrease in diarrhea duration and stool frequency, especially in those who started the intervention treatment within the first 24 h of symptom onset No impact on vomiting nor on fever duration	Rotavirus positive patients: the shortest diarrhea duration among those patients belonging to the intervention group Adenovirus positive patients: no influence on symptom duration *E. histolytica*: no influence on symptom duration

**Legend**: *B. bifidum*—*Bifidobacterium bifidum*; *B. breve*—*Bifidobacterium breve*; *B. lactis*—*Bifidobacterium lactis*; *B. infantis*—*Bifidobacterium infantis*; *B. longum*—*Bifidobacterium longum*; *B. mesentericus*—*Bacilus mesentericus*; CFU—colony forming units; *C. butyricum*—*Clostridium butyricum*; *C. difficile*—*Clostridium difficile*; *E. Coli*—*Escherichia Coli*; *E. faecalis*—*Enterococcus faecalis*; *E. histolytica*—*Entamoeba histolytica*; FOS—fructo-oligosaccharides: IV—intravenous, *L. acidophilus*—*Lactobacillus acidophilus*; *L. bulgaricus*—*Lactobacillus bulgaricus*; *L. casei*—*Lactobacillus casei*; *L. helveticus*—*Lactobacillus helveticus*; *L. paracasei*—*Lactobacillus paracasei*; *L. plantarum*—*Lactobacillus plantarum*; *L. rhamnosus*—*Lactobacillus rhamnosus*; *L. reuteri*—*Lactobacillus reuteri*; LF—lactose-free formula; ORS—oral rehydration solution; RCT—randomized controlled trial; *S. boulardii*—*Saccharomyces boulardii*; *S. thermophilus*—*Streptococus thermophilus*; Zn—zinc.

## 4. Discussion

The latest position paper of the Working Group on Probiotics and Prebiotics of the ESPGHAN, published in 2022, provides weak recommendations for the use of *L. rhamnosus* and *S. boulardii* in the management of acute pediatric gastroenteritis, based on low evidence [68]. An even lower level of evidence sustained the administration of *L. reuteri* for the same purpose, whereas a recommendation was issued against the use of *Bacillus clausii* and against the combination of *L. reuteri* and *L. helveticus* in childhood diarrhea, similarly to their previous publication [18,81]. The authors have provided no specific advice for diarrhea with an established etiology and have also mentioned the potential impact of rotavirus vaccine coverage in influencing their analysis of previously published data [81].

This review tried to add novelty to the existing literature data by assessing the impact of probiotic and synbiotic use upon symptom duration, severity and immune response in both viral and bacterial gastroenteritis in children. Most of the studies conducted so far which assessed the impact of probiotic use upon childhood enteric infections have focused solely on rotavirus diarrhea and provided the basis for two previous meta-analyses [82,83], as rotavirus still remains the leading cause of diarrhea-associated complications, especially in underdeveloped countries [84,85]. However, norovirus seems to overcome rotavirus as the etiological diarrheic agent which most commonly requires medical care in children, in those countries with high anti-rotavirus vaccination coverage, such as the United States [86]. Several theories have emerged regarding the possible inhibition of replication exerted by probiotics upon enteric viruses, and probiotic supplementation has been recommended for prevention of rotaviral diarrhea, as well as for diminishing symptom duration [82,85]. As previously presented and detailed in Table 1, Table 2, Table 3 and Table 4, several strains have proven their benefits in rotavirus-associated diarrhea, including individual strains, as well as combined probiotic preparations. The majority of these studies involved supplementation with either *L. rhamnosus* or *S. boulardii*, and combined suspensions included at least one of these two strains, which still represent the most studied and validated probiotics in the pediatric literature [18,87]. One study which analyzed a combined probiotic product was omitted from Table 4, due to the lack of information regarding the exact content of the product used, as only a commercial name was provided in the manuscript abstract and main text [88]. However, very few studies separately analyzed the impact of probiotic use upon recovery from bacterial diarrhea [23,30,59]. This might be explained by the small study populations included within randomized controlled trials, and the even more size-limited sub-cohorts in which a bacterial etiology was identified. Experimental research studies have suggested that probiotics such as *S. boulardii* and several *Lactobacillus* strains show antimicrobial effects, but with limited sensitivity against the analyzed pathogens [89,90].

On the other hand, several studies have evaluated the impact of probiotic strains such as *L. reuteri* or of a product consisting of *B. lactis*, *L. rhamnosus*, and *L. acidophilus* on diarrhea severity and duration, in spite of not reporting any information regarding the source of infection, which might have greatly influenced the analyzed outcome parameters [91,92,93]. In similar fashion, one Indian study asserted that *S. boulardii* reduces time until resolution of watery stools and duration of diarrhea after 5 days of treatment, but conducted no separate etiology-based evaluation, due to the very limited number of cases in which the source of diarrhea (rotavirus/*Vibrio cholerae*) was known [94]. Synbiotics containing FOS or inulin might also positively influence childhood gastroenteritis outcome, but there is insufficient data to assess the true impact of their use in particular infectious settings [95,96]. Consequently, the most recently published Cochrane systematic review underlines that there is insufficient data to allow pathogen-specific analysis of the role of probiotic supplementation in acute gastroenteritis, due to the paucity of the studies which have identified etiological agents of diarrhea or have conducted a separate statistical evaluation, focused on a certain pathogen [97]. Furthermore, the question of adequate, safety/efficacy balanced dosage of even the most widely used strains still remains into place and experts in the field suggest conducting trials comparing administration of different dosages of the same/various strains [98].

It is also worth mentioning that most of the studies conducted so far on the matter of probiotic efficacy in acute pediatric gastroenteritis included hospitalized children, and a very limited number of them were performed in outpatient settings or included follow-up of patients’ outcome after discharge from the hospital [30,31]. A decrease in the duration of diarrhea in relation to *L. reuteri* use was reported within a Turkish study performed in an ambulatory care unit [92]. Still, as represented in Table 1, Table 2, Table 3 and Table 4, most of the studies which evaluated pathogen-particular benefits of probiotics have only evaluated their efficacy after 5–7 days of administration.

Recent literature data supports the administration of probiotics also as a prophylactic measure against several enteric infections. Two studies conducted on a murine model showed that *S. boulardii* can effectively inhibit tissue inflammation and bacterial translocation, which provide protection against *Salmonella* [99,100]. Prevention of rotavirus gastroenteritis seems on the other hand to be enhanced by *L. rhamnosus* administration, especially in hospitalized children [101], as well as by supplemented formula feeding, containing strains such as *Bifidobacterium animalis subspecies lactis BB12* alone/in combination with *S. thermophilus* [102]. *L. acidophilus* also enhanced protection against rotavirus, according to a meta-analysis conducted by Di et al. [103]. *L. reuteri* on the other hand has been regarded as a possible prophylactic agent against diarrhea in pre-school children [104], helping maintain the integrity of the intestinal mucosa epithelial barrier, offering protection against its disruption, often caused by enterotoxigenic *E. coli* infection [105]. Moreover, *L. plantarum* interferes with binding of enteropathogenic *E. coli* to the intestinal epithelial cells [106]. The results obtained among in vitro studies still require confirmation among large-cohort studies and as Depoorter et al. suggest in their review, there is overall insufficient proof to routinely recommend probiotics for prevention of acute gastroenteritis in children [107].

## 5. Conclusions

Rotavirus infection remains the most frequently evaluated etiology in relation to probiotic efficacy, with multiple studies proposing a decrease in its related diarrhea and hospitalization duration with widely used probiotics such as *S. boulardii*, *L. reuteri* and *L. rhamnosus*, as well as synbiotic products containing at least one of these three strains, as complementary treatment to supportive therapy. There is, however, insufficient data on the impact of probiotics in relation to rotavirus immunization status or their influence on the diarrhea episodes treated in outpatient settings. Clinical reports are scarce regarding potential probiotic benefit in bacterial diarrhea, but some strains have been shown to inhibit in vitro growth of certain microbial pathogens. Future studies, conducted on larger cohorts, are warranted to assess the individual and additive effects of probiotic strains on outcome of pediatric gastroenteritis with identified causative agents.

## Data Availability

Not applicable.
